# Biocomposites of Low-Density Polyethylene Plus Wood Flour or Flax Straw: Biodegradation Kinetics across Three Environments

**DOI:** 10.3390/polym13132138

**Published:** 2021-06-29

**Authors:** Anna K. Zykova, Petr V. Pantyukhov, Elena E. Mastalygina, Christian Chaverri-Ramos, Svetlana G. Nikolaeva, Jose J. Saavedra-Arias, Anatoly A. Popov, Sam E. Wortman, Matheus Poletto

**Affiliations:** 1 Department of Biological and Chemical Physics of Polymers, Emanuel Institute of Biochemical Physics of Russian Academy of Sciences, Kosygina st. 4, 119334 Moscow, Russia; zykovaak@yandex.ru (A.K.Z.); p.pantyukhov@gmail.com (P.V.P.); elena.mastalygina@gmail.com (E.E.M.); anatoly.popov@mail.ru (A.A.P.); 2 Laboratory “Advanced Composite Materials and Technologies”, Department of Innovative Materials and Technologies, Plekhanov Russian University of Economics, Stremyanny per. 36, 117997 Moscow, Russia; 3 Departamento de Física, Facultad de Ciencias Exactas y Naturales, Campus Omar Dengo, Universidad Nacional, Calle 9, Avenidas 0 y 1, 40101 Heredia, Costa Rica; ccramos@una.ac.cr (C.C.-R.); snikolaeva17@gmail.com (S.G.N.); jsaavedr@una.ac.cr (J.J.S.-A.); 4 Department of Agronomy and Horticulture, University of Nebraska–Lincoln, 279 Plant Sciences Hall, Lincoln, NE 68583-0915, USA; swortman@unl.edu; 5 Postgraduate Program in Engineering of Processes and Technologies (PGEPROTEC), Exact Sciences and Engineering, Central Campus, University of Caxias do Sul (UCS), Caxias do Sul 95070-560, Brazil

**Keywords:** low-density polyethylene, lignocellulosic fillers, wood flour, flax straw, biocomposites, biodegradation

## Abstract

The purpose of this study was to assess the potential for biocomposite films to biodegrade in diverse climatic environments. Biocomposite films based on polyethylene and 30 wt.% of two lignocellulosic fillers (wood flour or flax straw) of different size fractions were prepared and studied. The developed composite films were characterized by satisfactory mechanical properties that allows the use of these materials for various applications. The biodegradability was evaluated in soil across three environments: laboratory conditions, an open field in Russia, and an open field in Costa Rica. All the samples lost weight and tensile strength during biodegradation tests, which was associated with the physicochemical degradation of both the natural filler and the polymer matrix. The spectral density of the band at 1463 cm^−1^ related to CH_2_-groups in polyethylene chains decreased in the process of soil burial, which is evidence of polymer chain breakage with formation of CH_3_ end groups. The degradation rate of most biocomposites after 20 months of the soil assays was greatest in Costa Rica (20.8–30.9%), followed by laboratory conditions (16.0–23.3%), and lowest in Russia (13.2–22.0%). The biocomposites with flax straw were more prone to biodegradation than those with wood flour, which can be explained by the chemical composition of fillers and the shape of filler particles. As the size fraction of filler particles increased, the biodegradation rate increased. Large particles had higher bioavailability than small spherical ones, encapsulated by a polymer. The prepared biocomposites have potential as an ecofriendly replacement for traditional polyolefins, especially in warmer climates.

## 1. Introduction

Very much attention of scientists nowadays has been focused on the research and development of new composites based on polymeric materials, which can be disposed in a safe way (i.e., biocomposites). According to Brebu (2020) [1], environmental degradation of polymeric materials is caused by a combination of physical, chemical, and biological processes. Temperature (thermal degradation), air (oxidative degradation), moisture (hydrolytic degradation), microorganisms (biological degradation), light (photo degradation), high energy radiation (UV irradiation), chemical agents (chemical corrosion), and mechanical stress are among the dominant factors.

The majority of the investigations are devoted to structure and properties of polymer blends or composites based on environmentally friendly polymer matrices, such as polylactide (PLA) [2,3], polyhydroxybutyrate (PHB) [4], polyhydroxybutyrate-co-polyvalerate (PHB-V) [5], and poly-ε-caprolactone (PCL) [6]. However, there has been another approach in order to overcome the biggest disadvantage of such matrices: very high cost. Some researchers consider hybrid materials based on synthetic matrices such as low density polyethylene (LDPE) [7,8], linear low density polyethylene (LLDPE) [9] and high density polyethylene (HDPE) [10], isotactic polypropylene (iPP) [11], natural rubber [12], or thermoplastic starch (TPS) [13,14] with natural fillers. This concept has several advantages including usage of agricultural and industrial waste as fillers, such as wood flour [15], cellulose [16], flax straw [17], banana [18], hemp, kenaf, wheat [19], rice [20], corn fibers [21], and marine residues of animal origin (e.g., chitin from shrimp shell) [3].

The effect of environmental conditions on biodegradation of polymeric materials were under the discussion in several works. Abiotic (temperature, soil moisture, and soil physical–chemical properties) and biotic (microbiome) environmental factors were determined as the most dominant in the polymer biodegradation process [22,23,24,25,26]. Shabani and colleagues (2015) [27] developed and conducted the theoretical experiment in order to estimate the possibility of virgin LDPE biodegradation under the action of *Aspergillus niger* fungi dependent on various climate factors and changes. According to their findings, the most effective LDPE biodegradation occurs in several parts of the world, including south western Russia, central and eastern Argentina, Uruguay, southern Brazil and eastern United States [27]. The conclusions were made based on the forecasting computer model, which determined the most favorable locations for *A. niger* growth over the next 90 years, taking into account predicting algorithms of future climate changing [27]. Jakubowicz et al. (2006) [28] explored the effect of air humidity, water, and compost environment on degradation rate. It was concluded that moisture had a significant accelerating effect on the thermal oxidation process. Possibly, this can be explained by hydrolysis of esters in the humid air.

Hoshino et al. (2010) [29] compared the biodegradation of several biodegradable polymers, including poly-(3-hydroxybutylate-valerate) (PHB/V), poly-(ε-caprolactone) (PCL), poly-(butylene succinate) (PBS), poly(butylene succinate and adipate) (PBSA), and poly-lactide (PLA) dependent on climate conditions of Japan. Temperature was strongly correlated with biodegradation of PHB/V, PCL, PBS, and PBSA, and precipitation was correlated with biodegradation of PCL and PBSA. Soil moisture is likely a better predictor of biodegradation given that rainfall can have negligible effects on soil moisture if the soil is already at or near field capacity. There was also a correlation between biodegradation and total soil nitrogen, but total carbon, pH, and texture were less significant. In this study, we investigated the biodegradability of composites based on LDPE and plant-based fillers including wood flour and flax straw. These biocomposites could have application as the basis for agricultural mulching films, molded goods, or packaging for consumer goods. This type of biocomposites has several advantages, including the low cost of production, usage of standard equipment, and simplicity of processing. In addition, the byproducts from agriculture, woodworking, textile, and food industries can be sourced locally and inexpensively and used as fillers.

Data on the thermal, mechanical, and other properties of the initial biocomposites were published earlier [30] and partly mentioned in the current study. It was reported that the size of filler particles had an influence on the kinetics of oxidative degradation and water absorption in biocomposites with FS. The primary aim of this investigation was to compare biodegradation rates of biocomposites between two diverse locations characterized by differences in climate and soil. This study also aims to evaluate how the lignocellulosic filler type (wood flour or flax straw) and particle size are affected by climate and soil conditions during biodegradation.

## 2. Materials and Methods

### 2.1. Materials

Wood flour (WF) and oil flax straw (FS) used in this study were provided by Novotop and Kostroma State University from Russia, respectively. Both fillers contain cellulose (WF 47%, FS 53%), lignin (WF 20%, FS 15%), hemicellulose (WF 32%, FS 11%), proteins (WF 0.3%, FS 6%), and fats (WF 0.4%, FS 3%) (Zykova et al., 2017).

Low-density polyethylene (LDPE 15803-020, MFI/190 °C/2.16 kg = 2 g/10 min, density = 0.92 g/cm^3^, T_m_ = 108 °C, χ = 26%) was supplied by Kazanorgsintez (Kazan, Russia).

### 2.2. Composite Preparation

FS stems were stripped of seeds. Both WF and FS were dried in air-circulating oven LOIP LF-120/300-VS2 (Saint Petersburg, Russia) at 105 °C for 3 h. Then, they were crushed by a rotary mill Vilitek DM-6 (Moscow, Russia) and filtered through a sieve set Matest A059-02KIT (Arcore, Italy) and laboratory sieves with a mesh of 80, 140, and 200 microns. Four fractions of each filler were collected from the sieves for the further work (0–80, 80–140, 140–200, and 0–200 µm).

The mixing of the polymers with the fillers was performed with 70% wt. of LDPE and 30 wt.% of filler (WF or FS). Plasti Brabender Plasti-Corder Lab-Station (Duisburg, Germany) with twin screw cam mixer was employed for making composites. The mixing temperature was 140 °C, and screw speed was 30 rpm. Then, the obtained pieces of composites were pressed for 3 min by a hydraulic press VNIR PRG-1–10 (Moscow, Russia) under the pressure of 7 kN with the plates heated to 135 °C. In order to form a fine crystalline structure, the obtained hot films were immediately immersed to the cool (10 °C) water. As a result, round films with the diameter of 70 mm and thickness of 150–200 µm were obtained to be used in biodegradation test.

### 2.3. Measurements

The biodegradation tests were carried out in a natural field soil and a soil mix (ASTM D 5988-12). The soil mix included equal parts of sand, horse manure, and garden soil, consistent with an approach previously used [7,8]. The soil mix was held for 2 months at 20 ± 3 °C with humidity maintained at 70% by watering and stirring. The film samples were placed vertically in the soil (30 cm depth) and carried out for 20 months with periodical inspections. Differences in appearance, mass, chemical composition, and degradation of the composites were analyzed.

Biodegradation was assessed in three different environments including:

1. Prepared soil mix under laboratory conditions (constant temperature (23 ± 3 °C) and constant humidity (60%)).
2. Prepared soil mix under ambient field environmental conditions in Moscow region, (Kubinka, Moscow region, Russia). A natural soil layer was removed (depth of 30 cm) and replaced with the prepared soil mix. There was no barrier between natural soil and the prepared soil mix.
3. Natural soil (not prepared soil mix) under ambient field environmental conditions in an experimental field at the Universidad Nacional (Heredia, Costa Rica) [30].

Moscow region has continental climate with expressed differences in temperature between summers and winters. The average annual temperature is +6 °C, but in February it is −12 °C and in July +20 °C. About 650 mm of atmospheric precipitation falls in Moscow region per year.

Heredia has a tropical savanna climate. The differences in the temperature between seasons are not clearly expressed (annual average temperature is +22 °C), but the precipitation has significant differences between dry winter and humid summer. The annual atmospheric precipitation is about 2000 mm.

The prepared soil mix (used in the laboratory conditions and field in Moscow region) and the natural Costa Rican soil were analyzed for chemical properties (Table 1). The soil mix was characterized by greater organic matter (138 g kg^−1^) and nitrate (728 mg kg^−1^) compared to the Costa Rican field soil (total organic C = 33.4 g kg^−1^; nitrate = 2 mg kg^−1^), and pH was similar between them (prepared soil mix = 6.5; Costa Rica soil = 6.2).

Optical microscopy (Carl Zeiss Axio Imager Z2M with AxioVision ver. 4.7.1, magnification 50× and 200× in transmitted and reflected light) was employed to find differences between initial samples and the same samples after soil immersion for 20 months.

Mechanical properties of materials before and after burial were investigated according to ISO 527–1:2012 via universal testing machine Devotrans DVT GP UG (Istanbul, Turkey). The samples were stretched to failure at a temperature of (22 ± 2) °C, and crosshead velocity was 0.25 mm/min. The dimensions of the samples: 70 mm × 10 mm × 0.15 mm, effective length—40 mm. The data for 7 samples were averaged. Differences among means were determined using 95% confidence intervals.

Chemical properties of materials before and after burial were studied on a FT-IR spectrometer Perkin Elmer Spectrum 100 (Waltham, MA, USA) at a temperature of (22 ± 2) °C in the range of wave numbers 4600 ≤ ν ≤ 650 cm^−1^ by a method of frustrated total internal reflection. Intensities of several peaks referring to polymer oxidation process (i.e., carbonyl group), microbiological colonization, and polymer degradation were determined, and its dynamics during biodegradation tests were detected.

All laboratory tests were carried out using scientific equipment at the Center of Shared Usage «New Materials and Technologies» at Emanuel Institute of Biochemical Physics and Joint Research Center at Plekhanov Russian University of Economics.

## 3. Results

**Figure 1:**
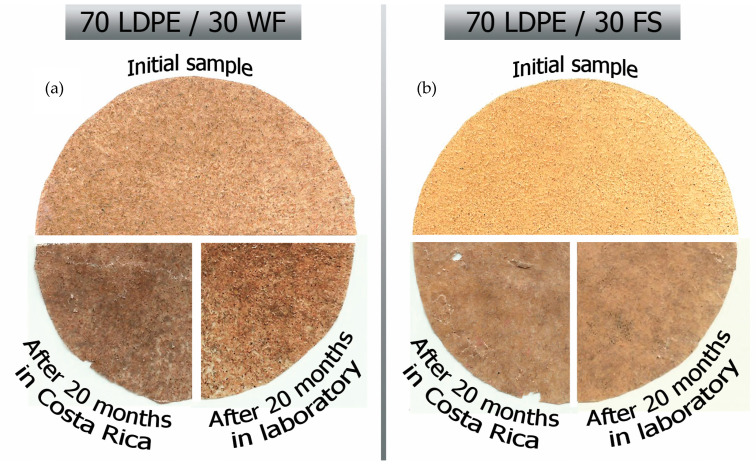
Scanned pictures of biocomposites before and after biodegradation test in the laboratory and in Costa Rica for WF (**a**) and FS (**b**).

**Figure 2:**
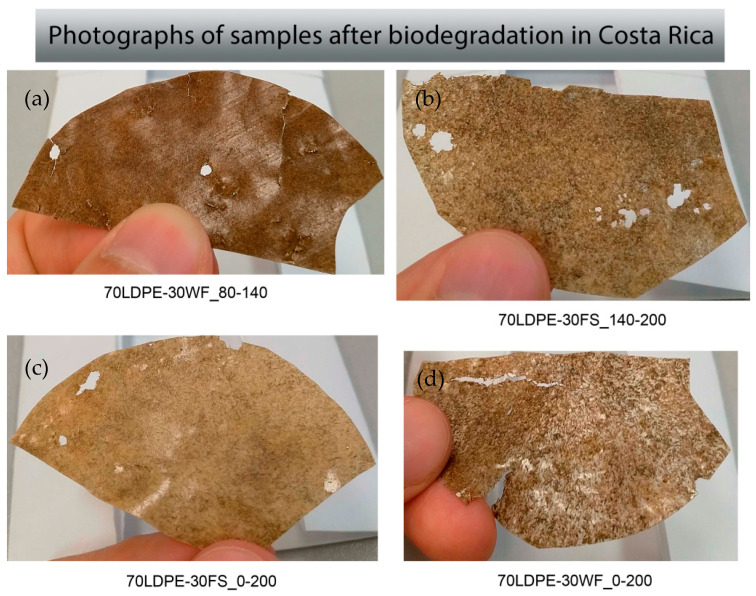
Photographs of damaged of (**a**) 70LDPE-30WF_80-140, (**b**) 70LDPE-30FS_140-200, (**c**) 70LDPE-30FS_0-200, and (**d**) 70LDPE-30WF_0-200 samples after 20 months of biodegradation in Costa Rica.

The initial color of composites with WF was darker than FS (Figure 1), and both composites became darker after soil burial. The samples after soil burial in Costa Rica had mechanical damage likely due to disturbance from plant roots, insects, and animals (Figure 2). Filler particles (wood flour and flax) near the material surface vanished during soil burial leaving only a spongious polyethylene layer. Film samples of biocomposites with fraction 0–80 μm exhibited a visibly smoother surface both before and after soil burial.

The trends of degradation tests in different climatic conditions were partly discussed earlier [30]. The weight loss was greater for FS composites than that of the WF composites (Table 2). The biodegradation rate in Costa Rica soil and field conditions was greater than in Russia field conditions with prepared soil mix (Table 2). The enhanced biodegradation rate of composites in Costa Rica can be associated with the higher temperature and humidity than the others ambient tested. In addition, the dependence of weight loss on the filler fraction was found. The larger the filler particles were, the faster the weight loss during biodegradation was. The complex fractions (0–200 µm) of WF composites lost even more weight than the biggest fraction (140–200 µm). However, the FS complex fractions demonstrated the average value between all the fractions. Samples buried in Costa Rica had many holes, cracks, and torn edges, which suggests plant roots and soil meso- and macro-fauna could have contributed to physical degradation (Figure 2).

**Figure 3:**
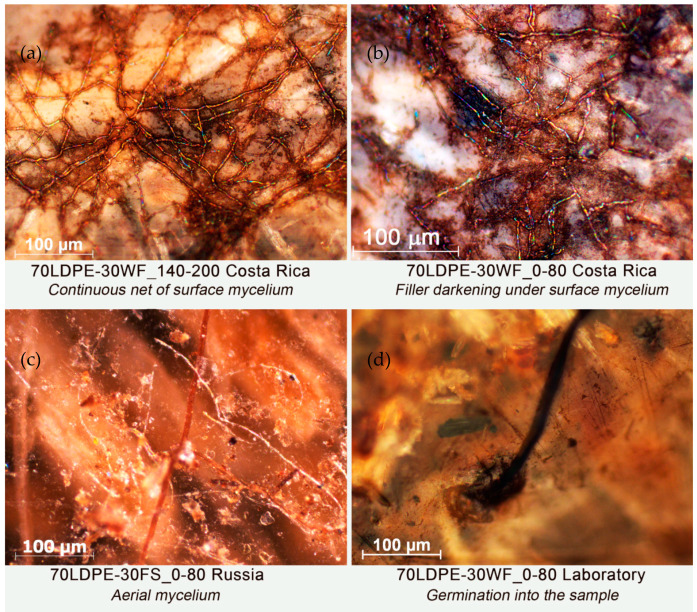
Microphotographs of (**a**) 70LDPE-30WF_140-200 Costa Rica, (**b**) 70LDPE-30WF_0-80 Costa Rica, (**c**) 70LDPE-30FS_0-80 Russia, and (**d**) 70LDPE-30WF_0-80 laboratory samples after biodegradation test. Reflected light, magnification 200×.

The microphotographs of the samples after burial in soil are presented in Figure 3. Cracks, caverns, and erosions were detected in all samples. The biodeterioration of the filler particles was found in the majority of the samples, which provides the confirmation of microbial colonization into the composite. In several samples, fungal hyphae formed a transparent net of mycelium. The hyphae of fungi and humidity have the potential to penetrate a composite and inflict great damage; in particular, the cellulose content of the lignocellulosic filler is a source of nutrition for the microorganism [31,32].

**Figure 4:**
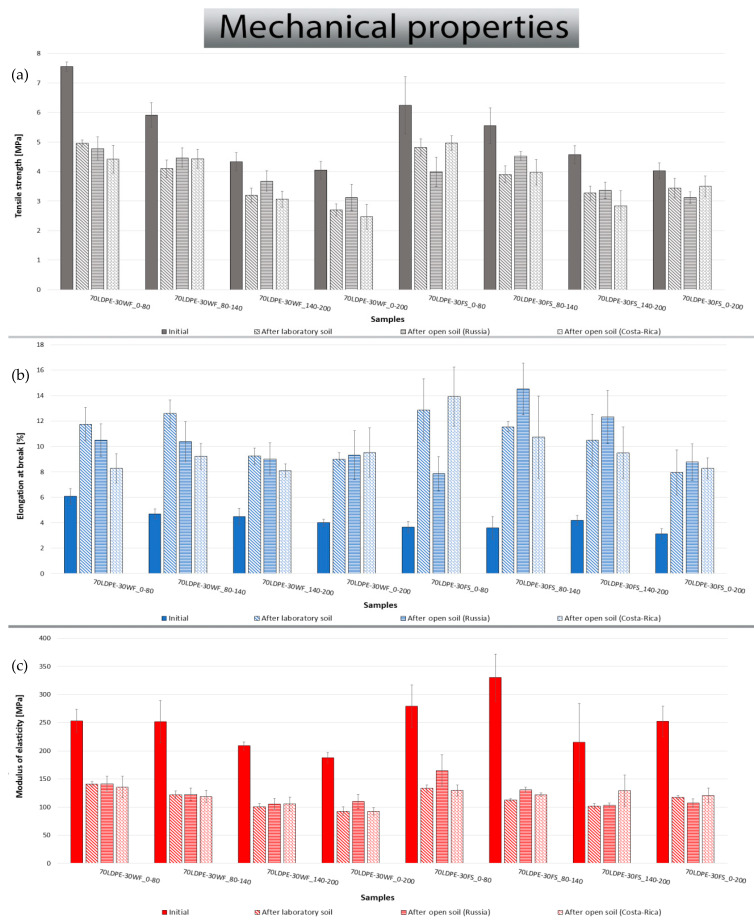
Tensile strength (**a**), elongation at break (**b**) and modulus of elasticity (**c**) of biocomposites before and after biodegradation tests.

All the mechanical properties of initial samples and samples after biodegradation under three environments are collected and presented in Figure 4. The mechanical characteristics of initial biocomposites are acceptable for the most part of possible applications. After biodegradation, the tensile strength of all the composites decreased by an average of 25%, elongation at break increased by an average of 185%, and elasticity modulus decreased by 55%, which can be associated with the biodegradation of lignocellulosic filler which may contribute the reduce composite stiffness. The decrease in the elasticity modulus and consequent increase in the elongation at break might be associated with the absorption of water by the lignocellulosic filler, due its hydrophilic nature, which contributes to the biodegradation and plasticity of the composite [33]. The tensile strength of initial biocomposites depends on the nature of the filler and its particle size. However, particle size appears to be a more pronounced effect. The lower the particle size is, the higher the tensile strength of biocomposites on its base is. Comparing the two fillers, there was no obvious difference in the reduction of tensile strength after biodegradation. Significant differences were not detected in the mechanical properties after biodegradation under different environments. This can be explained by the fact that only the most defect-free parts of the samples were cut away for the mechanical tests, and the environments differ by the size and quantity of physical defects in the sample.

**Figure 5:**
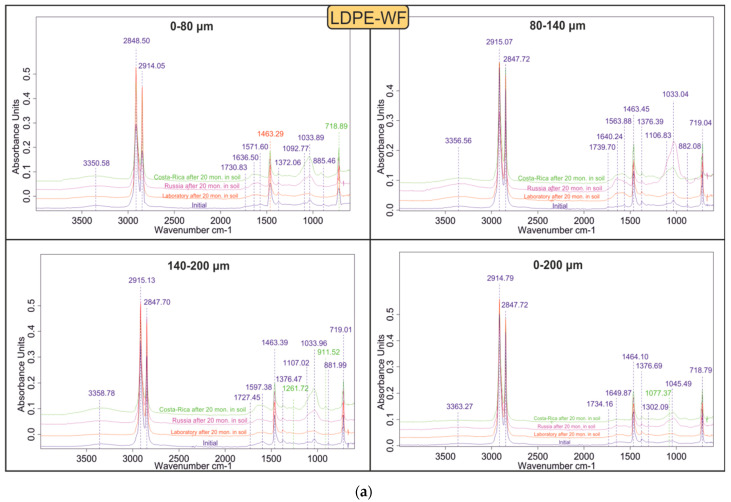

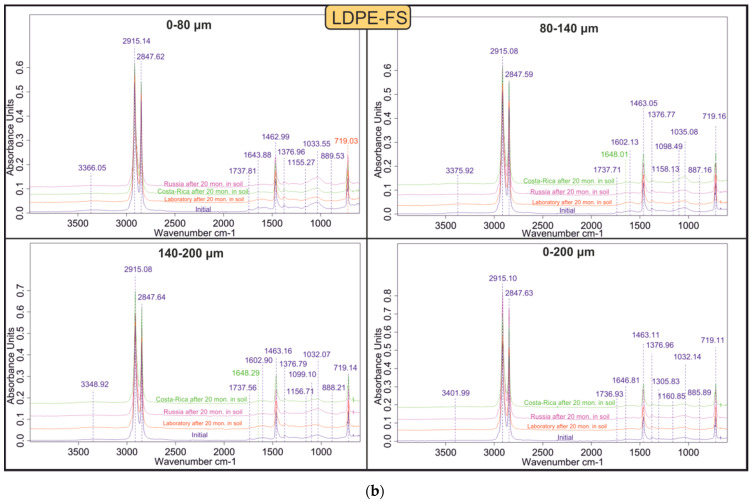
FTIR spectra of biocomposites with wood flour (**a**) and flax straw (**b**) according particle size used. Initial samples, samples after soil tests for 20 months in laboratory conditions, in Russia, and in Costa Rica.

Changes in chemical composition of the samples were analyzed by FTIR-spectroscopy (ATR method). The FTIR spectra from the lignocellulosic fillers are previously published [34]. FTIR spectra of LDPE/WF composites and LDPE/FS (initial samples and samples after soil tests for 20 months) are shown in Figure 5a,b, respectively. All the spectra were normalized by spectral intensity of the band at 2915 cm^−1^. Composites buried in different soil conditions had different changes in chemical composition. FTIR-spectra of initial LDPE/WF and LDPE/FS composites have an absorbance peak at 1030 cm^−1^, which corresponds to the band of skeletal vibrations of C-O groups in cellulose. The broad peak around 3500–4000 cm^−1^ is assigned to O-H stretching vibrations from -OH groups of lignocellulosic substances. The absorption band observed at 1710 and 1740 cm^−1^ is indicative of the presence of the lignin component in the filler. The spectra of all initial samples have absorbance in the region of 1550–1650 cm^−1^ due to C=C vibrations in aromatic rings of lignin.

**Figure 6:**
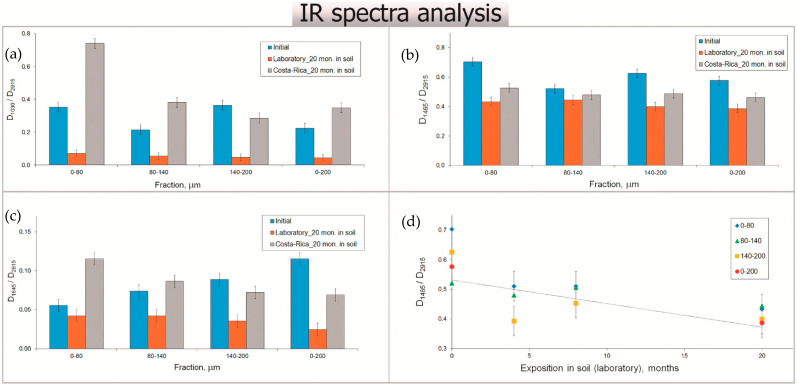
FTIR data analysis. The share of bands height at 1030 (**a**), 1465 (**b**), and 1645 cm^−1^ (**c**) to reference band at 2915 cm^−1^. The reduction kinetics of band at 1465 cm^−1^ during the biodegradation test (**d**).

After a recovered soil test under laboratory conditions, the intensity of peaks corresponding to functional groups of lignocellulosic components decreased. The spectral density of band 1030 cm^−1^ significantly decreased after soil burial under lab conditions compared to these bands for initial samples (Figure 6). This behavior may be attributed to the chain scission of crystalline and amorphous cellulose chains during biodegradation [35]. From the FTIR spectra of composite samples after soil burial in Costa Rica, absorption band 1030 cm^−1^ grew compared to initial samples. This was most evident for the composites with the smallest fraction of WF particles (LDPE/WF 0–80 μm). This composite had the most intensive biofouling by fungal mycelium on the surface according to microscopy analysis [36]. The band increases at 1030 cm^−1^ for the sample from Costa Rica can be associated with the increase in chitin molecules contained in fungal cell walls [36].

According to biodegradability test results, LDPE as a part of LDPE/WF composites underwent transformations of molecular structure. We observed a change in optical density ratio of the peaks at 2915 cm^−1^ (CH asymmetrical stretching vibrations) and 1463 cm^−1^ (C-H scissoring bending vibrations) [37,38]. The band 1463 cm^−1^ assigns to quantity of CH_2_-groups in polyethylene chains (Figure 6). The primary mechanism for the biodegradation of high-molecular-weight polymer is the oxidation or hydrolysis by enzyme creating functional groups that improve the hydrophilicity [39]. Consequently, the main chains of polymer are degraded resulting in polymer of low molecular weight and more accessible for further microbial assimilation [39].

## 4. Discussion

The films of prepared biocomposites with the smallest fraction (0–80 µm) had a smoother surface than the others. Small particles of the fillers were better encapsulated by the LDPE polymer matrix than the larger ones; apparently, this inhibits the biodegradation of composites with small particles (supporting results to follow). The darkening of the samples after soil burial can be explained by several factors: blackening of filler particles; refraction of rays in the holes and cracks in the biocomposite; diffusion of soil into the formed cavities inside the biocomposite; and oxidation of the polymer matrix. The importance of temperature relative to soil nitrogen was further evidenced by differences between Costa Rica and the laboratory environment; the temperature was greater and soil nitrate was lower in Costa Rica compared to the lab, yet biodegradation was greater in Costa Rica (Table 2).

The weight loss of FS composites was greater than that of the WF composites (Table 2). This can be explained by both the chemical composition of fillers and the shape of filler particles. Flax straw has greater cellulose, protein, and fat content compared to wood flour. It was discovered that composites with bigger particles are more prone to biodegradation than those with small particles perhaps because small particles are better encapsulated by the synthetic polymer matrix. Moreover, bigger filler particles had a greater length-to-diameter ratio and connected with each other forming a “net”, which allowed for the penetration of microorganisms. This is consistent with previous work [7], where it was found that as the length-to-diameter ratio of fillers increased, the weight loss rose.

The biodegradation process in Costa Rica was a lot quicker than in Russia (Table 2). This is likely due to the warmer temperatures and greater precipitation in Costa Rica and highlights the importance of weather relative to soil properties in predicting biodegradation rates. Hoshino et al. (2001) [29] found strong correlations between soil temperature and biodegradation of several biobased polymers, and soil nitrogen was also a strong predictor of biodegradation. Kim et al. (2006) [35] also report that low soil temperature can promote a slow rate of hydrolysis of lignocellulosic material filled in polymer composites. In this study, biodegradation was greater in Costa Rica despite substantially lower soil nitrate and organic matter, which suggests that the soil temperature and precipitation were more important drivers of degradation.

After biodegradation in Costa Rica, samples had many physical damages (Figure 2), which is an important initial stage of biodegradation. Because of abiotic reactions (oxidation, hydrolysis, photo, or thermal degradation) at the initial stage, polymer decreases its molecular weight, the film sample becomes more fragile and brittle, and it falls apart. At the second stage, biotic processes caused by microorganisms begin, up to full mineralization [40].

According to the results of the mechanical assay of initial composites, the nature of the filler has little effect on the tensile strength, but it depends on the particle size (Figure 4). Elongation at break depends both on the filler nature and dispersivity. Apparently, small particles interfere with the straightening of polyethylene macromolecules less than the large ones. It is likely that more elongated particles of FS (L/D ratio of FS = 6.7, L/D ratio of WF = 4.5) [41] inside randomly oriented films are more likely to interfere with the straightening of polyethylene macromolecules.

After biodegradation, mechanical properties of all the biocomposites changed significantly, they become more elastic but less durable. Results can be attributed to the biodegradation of filler particles that interfered with the straightening of polyethylene macromolecules at the elongation process. The biodegradation of some major components presented in the lignocellulosic fillers used may contribute to this result. The biodegradability of lignin and cellulose results from the hydrolytic depolymerization of these major lignocellulosic filler components to low-molecular-weight materials which then yield monomeric units [35]. The biodegradability of cellulose possibly involves random chain scission of the bonded β-1,4-glucosidic and that of lignin involves cleavage of the chains of the phenyl propane units [35]. As a result, the biodegradation of filler particles result in a more elastic composite, since lignocellulosic fillers are more rigid than polymer matrix and also contribute to restricting the flow of polymer chain segments from the matrix [42]. Formed voids contribute to a decrease in the stiffness of the composite and an increase in its elasticity. However, oxidation of the polymer matrix due to the action of microorganisms’ metabolites at the large composite surface area leads to a decrease in tensile strength. At the same time, the oxidation of polyethylene should be accompanied by enhanced rigidity and reduced elongation [8,43]; however, the effects of this process are less than the effects of macromolecules’ straightening.

The decrease in band 1030 cm^−1^ (Figure 6) after the biodegradation test was attributed to the destruction of WF compounds in the composites followed by the weight loss of the samples. Samples with bigger fractions had fungal damages, concentrated in filler particles. For the smallest fraction, a continuous network of mycelium was visible on the surface. It is likely that the increased band at 1030 cm^−1^ is related to the increased number of C-O groups in chitin molecules contained in fungal cell walls and consistent with previous research [36]. An increase in spectral density of the band at 1645 cm^−1^ (corresponding to N-H bending vibrations) indicates accumulation of proteinic materials [43] on the sample surface, which also suggests materials biofouling. The same behavior was also verified by Fabiyi et al. (2011) [37] in composites of HDPE/wood flour. In the process of soil burial, the ratio of D_1463_/D_2915_ decreased, which is evidence of polymer chain breakage with formation of CH_3_ end groups. The band intensity at wavenumber of 910 cm^−1^ increased after soil burial in Costa Rica, which could be explained by a formation of C=C bonds in the polymer chains.

**Figure 7:**
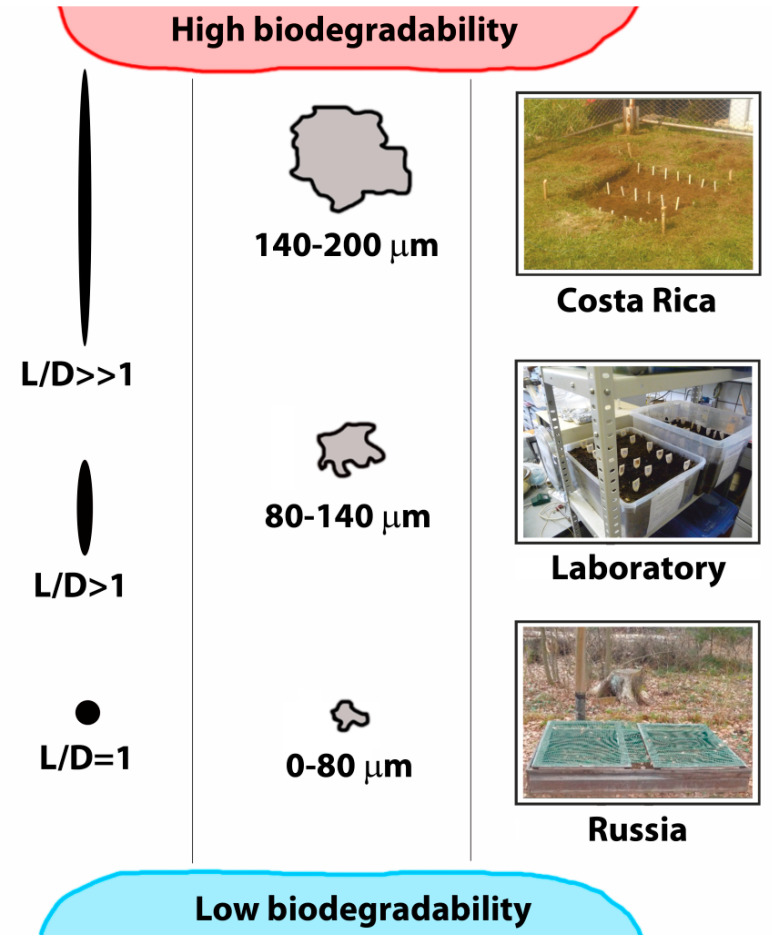
Scheme showing the effect of lignocellulosic filler size on the composite degradation.

Probably, the degradation of biocomposite materials begins from the adhesion of fungi and bacteria on the surface of film sample [44,45]. Lignocellulosic filler particles nearby the surface biodegrade first. Elongated particles with large diameters are more susceptible to biodegradation than the small spherical ones, as represented by the degradation scheme proposed in Figure 7.

During the biodegradation of lignocellulose, the metabolites of microorganisms are produced. Metabolites provoke the oxidative degradation of polymeric matrix, and then the microbiological processes take place. Physical destruction caused by roots of plants, insects, worms, animals, and temperature changes accelerates the degradation of polymer matrix because of higher accessibility of filler particles for microorganisms. In warmer climates, these processes are more intensive. The period of biodegradation of biocomposites in warmer climate is less, and even biocomposite materials with polyethylene may be biodegradable there. For that reason, the developed biocomposites may be positioned as the ecofriendly replacement for traditional polyolefins in countries with warm climate.

## 5. Conclusions

Across diverse environments, the weight loss during soil burial of biocomposites was greatest in Costa Rica, followed by laboratory conditions, and lowest in Russia. The highest soil humidity in Costa Rica contributed to accelerating the biodegradation rate due to the cellulose main chain scission. Therefore, the hyphae of fungi and humidity could penetrate into the composite and inflict great damage to the samples tested. The larger the size of particles in the biocomposite material was, the greater the weight loss rate after biodegradation was. Destruction of the polymer matrix was shown by the reduced ratio 1465/2915 cm^−1^ in IR spectra for all the samples after biodegradation. Tensile strength and elasticity modulus of all the samples were reduced after soil burial. The tested biocomposites demonstrated excellent potential for application in agricultural or packaging industries as ecofriendly alternatives to polyethylene films, especially in warmer climates where biodegradation rates may be higher.

## Figures and Tables

**Table 1:** Chemical properties of soil analyzed from Russia and Costa Rica.

Soil Property	Russian Soil Mix	Costa Rica Field Soil
Organic matter (g kg^−1^)	138	-
Organic carbon (g kg^−1^)	-	33.4
pH (1:1 soil:water)	6.5	6.2
Organic nitrogen (g kg^−1^)	-	2.8
Nitrate (mg kg^−1^)	728	1.6
Ammonium (mg kg^−1^)	21	3.6
Phosphorus (mg kg^−1^)	421	8
Potassium (mg kg^−1^)	856	417

**Table 2:** Weight loss of biocomposites in different climatic conditions.

Climatic Conditions	Time of Exposition, Months	Weight Loss, %							
		70 LDPE/30 WF				70 LDPE/30 FS			
		0–80	80–140	140–200	0–200	0–80	80–140	140–200	0–200
Laboratory conditions	0.5	0	0.2	0.8	1.3	4.8	7.2	8.61	6.2
	2	0.7	2.8	6.7	8.6	14.2	16.6	17.60	14.8
	4	4.2	8.0	12.2	12.9	16.6	18.8	19.94	17.4
	6	7.7	11.0	14.7	15.5	17.8	19.9	21.20	18.5
	8	11.4	13.6	16.6	17.6	18.8	20.8	22.04	19.4
	10	13.1	14.6	17.3	18.7	18.8	21.0	22.38	19.8
	12	13.3	14.9	17.5	19.0	18.8	20.9	22.38	19.3
	14	15.2	16.3	18.2	20.1	19.2	21.5	23.05	20.1
	16	15.5	16.5	18.7	20.4	19.4	21.5	23.14	20.2
	20	16.0	16.9	19.2	21.1	19.7	21.7	23.30	20.7
Open air, Russia	6	6.2	7.2	8.2	9.2	10.2	11.2	12.2	13.2
	10	6.5	7.5	8.5	9.5	10.5	11.5	12.5	13.5
	20	13.2	15.4	17.2	19.5	19.3	20.7	22.0	20.1
Open air, Costa Rica	2	5.2	6.1	8.4	21.9	17.5	18.9	21.3	17.1
	6	10.4	12.2	15.2	26.7	19.3	21.4	24.9	20.7
	12	28.4 *	18.1	20.5	30.5	22.1	24.7	28.6	24.9
	20	30.9 *	20.8	23.5	31.8	23.5	26.1	30.6	26.3

* Samples were physically damaged in soil.

## Data Availability

The data presented in this study are available on request from the corresponding author.
